# Understanding socioeconomic differences in metabolic syndrome remission among adults: what is the mediating role of health behaviors?

**DOI:** 10.1186/s12966-021-01217-5

**Published:** 2021-11-09

**Authors:** Liza A. Hoveling, Aart C. Liefbroer, Ute Bültmann, Nynke Smidt

**Affiliations:** 1 Department of Epidemiology, University of Groningen, University Medical Center Groningen, PO Box 30.001, 9700 RB Groningen, The Netherlands; 2 Netherlands Interdisciplinary Demographic Institute, PO Box 11650, 2502 AR The Hague, The Netherlands; 3 Department of Sociology, Vrije Universiteit Amsterdam, De Boelelaan, 1081 HV Amsterdam, The Netherlands; 4 Department of Health Sciences, Community and Occupational Medicine, University of Groningen, University Medical Center Groningen, PO Box 30.001, 9700 RB Groningen, The Netherlands

**Keywords:** Metabolic syndrome, Socioeconomic factors, Physical activity, Smoking, Alcohol drinking, Diet, Longitudinal studies, Mediation

## Abstract

**Background:**

Although the incidence of metabolic syndrome (MetS) strongly varies based on individuals’ socioeconomic position (SEP), as yet no studies have examined the SEP-MetS remission relationship. Our aim is to longitudinally assess the associations between SEP measures education, income and occupational prestige, and MetS remission, and whether these associations are mediated by health behaviors, including physical activity, smoking, alcohol intake and diet quality.

**Methods:**

A subsample (*n* = 16,818) of the adult Lifelines Cohort Study with MetS at baseline was used. MetS remission was measured upon second assessment (median follow-up time 3.8 years), defined according to NCEP-ATPIII criteria. To estimate direct associations between SEP, health behaviors and MetS remission multivariable logistic regression analyses were used. To estimate the mediating percentages of health behaviors that explain the SEP-MetS remission relationship the Karlson-Holm-Breen method was used. Analyses were adjusted for age, sex, the other SEP measures and follow-up time.

**Results:**

At the second assessment, 42.7% of the participants experienced MetS remission. Education and income were positively associated with MetS remission, but occupational prestige was not. The association between education and MetS remission could partly (11.9%) be explained by health behaviors, but not the association between income and MetS remission.

**Conclusions:**

Individuals with higher education more often experienced remission from MetS, mainly because individuals with higher education were more likely to have healthier behaviors. However, individuals with higher income more often experienced MetS remissions, regardless of their health behaviors. The occupational prestige of individuals was not associated with MetS remission.

**Supplementary Information:**

The online version contains supplementary material available at 10.1186/s12966-021-01217-5.

## Background

Metabolic syndrome (MetS) is a cluster of interrelated components, including abdominal obesity, elevated blood triglyceride, reduced blood high-density lipoprotein (HDL) cholesterol, elevated blood pressure, and elevated fasting blood glucose; these components increase the likelihood of developing cardiovascular diseases (CVD), and of all-cause mortality [1]. Of the adult European population, 10–30% suffer from MetS, and this number is expected to increase [2, 3]. Individuals with a low socioeconomic position (SEP) have a greater risk of developing MetS [4]. However, little is known about SEP differences in reversing MetS. Evidence suggests that individuals with a higher SEP who have any of the MetS indicators are more likely to reverse this to a normal level; in general, however, evidence is limited [5–7].

SEP is a multifaceted concept that refers to an individuals’ cultural, economic and social resources [8]. Cultural resources comprise behavioral values and norms, knowledge, attitudes, beliefs and skills related to health and health promotion, and in health research are usually operationalized by educational attainment. Economic resources refer to available material assets and are usually operationalized by income. Social resources are thought to facilitate access to healthcare and are usually operationalized by occupational prestige [8–10]. Given the multifaceted nature of SEP, examining which of its aspects are most strongly related to MetS remission can provide targets for the key resources involved in remitting the syndrome.

If SEP differences in remitting MetS exist, a better understanding of the mechanisms involved is crucial in order to identify ways to redress the differences. Health behaviors may constitute an important class of mediators, as large SEP differences are related to these behaviors. Individuals with a high SEP generally exhibit healthier behaviors, which are, in turn, important factors for remission of MetS [11–16]. However, as the influence of health behaviors on SEP differences in MetS remission is as yet unclear, investigating their role may reveal important mechanisms to act on.

**Fig. 1:**
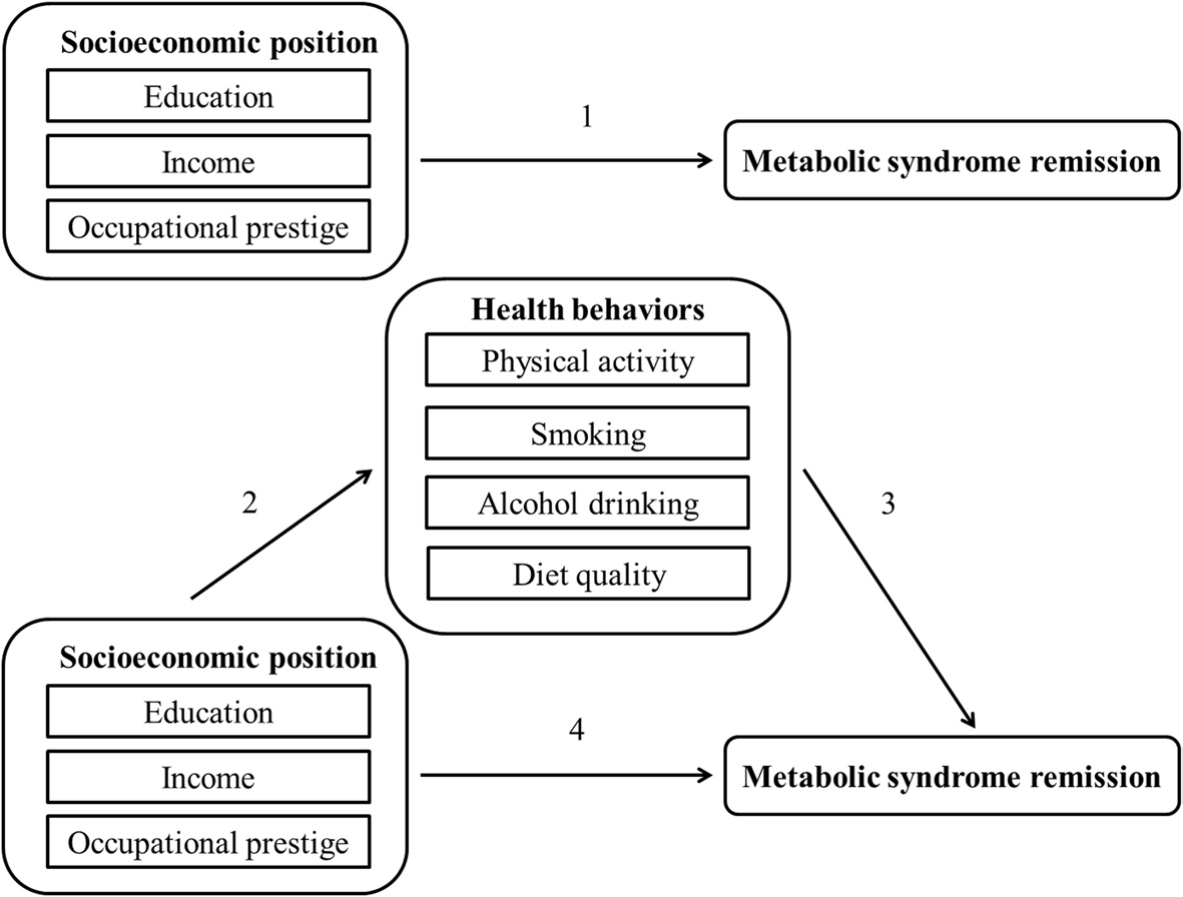
Graphical representation of direct associations between socioeconomic position and metabolic syndrome remission and indirect associations via health behaviors

The aim of the current study is to contribute to the literature in at least three ways. First, in this longitudinal study with a large sample of participants we will examine SEP differences in MetS remission. Second, we will examine the most pronounced indicator of SEP (education, income or occupational prestige) in relation to MetS remission. Third, we will examine the mediating role of health behaviors (physical activity, smoking, alcohol intake and diet quality) on SEP differences in MetS remission (Fig. 1).

## Methods

### Study design and sample

The study sample was derived from the Lifelines Cohort Study [17]. Lifelines is a multi-disciplinary prospective population-based cohort study examining in a unique three-generation design the health and health-related behaviors of 167,729 persons living in the north of the Netherlands. Lifelines employs a broad range of investigative procedures to assess the biomedical, socio-demographic, behavioral, physical and psychological factors that contribute to the health and disease of the general population, with a special focus on multi-morbidity and complex genetics. The study profile of Lifelines, and the recruitment and collection of the data are described elsewhere [17]. Baseline assessment, consisting of a physical examination, collecting blood and urine samples, interviews and self-report questionnaires, was conducted between 2006 and 2013. Participants were followed up approximately every 5 years with a physical examination involving drawing blood samples, collecting urine samples, and testing cognitive performance. In the current study, on average, 3.8 years elapsed between baseline assessment and second assessment.

The current study used a subsample of 24,458 participants aged 18 years and older, who had complete data for ≥70% of the variables needed for this study at baseline, and had MetS at baseline. Participants who were lost to follow-up upon second assessment (*n* = 6626), and for whom no MetS status could be determined based on the data of the second assessment (*n* = 1024) were excluded from the analyses. Finally, 16,818 (69%) participants were used for the analyses.

### Measures and procedures

#### Socioeconomic position

The independent variables defining SEP in this study were years of education, net household equivalized income and occupational prestige, all measured at baseline. *Educational level* was recoded into years of education, using the number of years it would take to complete each category by the fastest route possible (see Supplementary Table 1 for measurements of the relevant variables in the Lifelines Cohort Study) [18]. *Income* was recoded as the net household equivalized income, determined by dividing the midpoint of each participant’s  net income category by the square root of his or her household size [19]. The amounts were divided by 100; the model estimates thus show the difference in odds ratio (OR) of MetS for a 100-euro difference in household equivalized income. *Occupational prestige* was recoded from the International Standard Classification of Occupations 2008 (ISCO08) [20] to the continuous Standard International Occupational Prestige Scale 2008 (SIOPS08) [21] and divided by 10; the model estimates thus show the difference in OR of MetS for a 10-point difference in occupational prestige score. SIOPS08 is a continuous scale ranging from 0 to 100, indicating low to high occupational prestige [22].

#### Metabolic syndrome remission

*MetS* status (yes/no) was dichotomized and used as dependent variable. MetS was considered remitted when, according to the National Cholesterol Education Program’s Adult Treatment Panel III (NCEP-ATPIII), only up to two of the components were present upon second assessment [1]. The criteria are: 1) Waist circumference ≥ 102 cm in male or ≥ 88 cm in female; 2) Systolic blood pressure ≥ 130 mmHg, diastolic blood pressure ≥ 85 mmHg or use of blood pressure-lowering medication; 3) Triglycerides ≥150 mg/dL (1.7 mmol/l) or use of medication for elevated triglycerides; 4) HDL cholesterol < 40 mg/dL (1.0 mmol/L) in male or < 50 mg/dL (1.3 mmol/L) in female, or use of lipid-lowering medication; 5) Fasting blood glucose level ≥ 100 mg/dL (≥ 5.6 mmol/l), diagnosis of type 2 diabetes, or use of blood glucose-lowering medication. Medication use at baseline was classified according to the Anatomical Therapeutic Chemical coding scheme [23], and upon second assessment classified with a general question about current medication use (yes/no).

#### Health behaviors

Health behaviors were defined by total physical activity, smoking, alcohol intake, and diet quality, measured at baseline. *Physical activity* was measured using the SQUASH questionnaire, and dichotomized based on whether participants performed moderate to vigorous physical activity for at least 30 min per day (yes/no), at least 5 days per week [24, 25]. *Smoking* habits were categorized as never, former (i.e., smoked for a full year, but not a current smoker) or current (i.e., current smoker or smoked in the past month) smoker. *Alcohol intake* measured with the Food Frequency Questionnaire was categorized as no, moderate (i.e., one glass or less alcohol per day on average, without binge drinking (more than three glasses alcohol on 1 day for females and more than four glasses alcohol on 1 day for males)), or excessive (i.e., more than one glass alcohol per day on average, or binge drinking) alcohol intake [26, 27]. *Diet quality* was based on the Lifelines Diet Score (LLDS), which was in turn based on the 2015 Dutch Dietary Guidelines [28, 29]. In the Lifelines Cohort Study the LLDS was calculated per participant as the sum of positive and negative food group quintile scores (range 0–48) as described by Vinke et al. [28]. In the current study, as no participants had an LLDS of 0 participants were divided into three groups according to their LLDS: ‘poor’ diet quality (LLDS 1–16), ‘moderate’ diet quality (LLDS 17–32) or ‘high’ diet quality (LLDS 33–48).

#### Covariates

Age and sex at baseline and time between baseline and second assessment, were used as control variables in all models.

### Statistical analysis

Baseline characteristics concerning demographics, MetS indicators and health behaviors were described. We used multivariable logistic regression analysis, and multinomial- (for smoking status) or ordinal logistic regression (for alcohol intake and diet quality), controlling for age, sex, other SEP measures at baseline and time between baseline and the second assessment, to estimate the direct associations between SEP, health behaviors and MetS remission (Fig. 1, paths 1, 2 and 3). To estimate total-, direct- and indirect associations between SEP and MetS remission via health behaviors and the mediating percentages of the health behaviors, we used the Karlson-Holm-Breen (KHB) method [30]. We used the KHB method to decompose the total effects of the SEP variables on MetS remission in the non-linear models into the sum of the direct effects and indirect effects. The KHB method has two advantages over other mediation analyses. First, effects across nested non-linear models cannot be directly compared because regression coefficients and the error variance are not separately identified, this results in different error variances across such models that do not allow comparison of effects across models [31]. This problem of ‘rescaling’ of the error variance across nested models makes it impossible to simply examine the change in the effect of SEP on MetS remission after including the health behaviors as mediating variables that have an independent influence on MetS remission. The KHB method corrects for this rescaling and provides an estimate of how much each health behavior variable, conditional on the presence of the other health behaviors in the model, mediates the association between the SEP variables and MetS remission. Second, considering the multiple potential mediating health behaviors in the current study (including possibly correlated health behaviors), the KHB method provides the following advantages: 1) it decomposes the independent mediating effects of each individual health behavior (independent of correlation), making it possible to investigate differences among these effects and to determine the relative magnitude of each specific one; and 2) it calculates whether the change in the independent variable across models is greater than would be expected by chance. The results of all steps were presented as OR with 99% Confidence Intervals (CI). ‘Healthy’ behaviors were set as reference categories: physically active, never smoker, moderate alcohol intake and high diet quality. Participants were excluded if three or more MetS indicators were missing or, because they had provided information only on three or four indicators, we could not determine whether they had MetS. To impute missing values on the SEP measures and the health behaviors we used the Multiple Imputation by Chained Equation (MICE) method [32]. MICE was used and 10 datasets were created with 100 iterations for each dataset. We used auxiliary variables length and weight to give extra information about the incomplete values [33]. The imputation model included the independent variables, the covariates, the mediating variables, the dependent variables and the auxiliary variables.

Additionally, to assess whether changes in health behaviors between baseline and the second assessment affected the associations, we repeated the main analyses using a change score for the available health behaviors. Change scores were coded to indicate whether a participant’s specific health behavior remained the same, increased or decreased between baseline and the second assessment. Physical activity, smoking and alcohol intake were added to the main models; diet quality was not available upon second assessment, and was not added to the models.

To evaluate the robustness of the results, we performed sensitivity analyses. First, to assess the potential role of misclassification of medication use, we repeated the analyses for participants with MetS at baseline when medication had not been taken into account (*n* = 13,349). We also repeated analyses separately for participants who reported that they had used medication during the second assessment (*n* = 11,172), and for participants who indicated that they had not used medication during the second assessment (*n* = 4015). Secondly, we performed a complete case analysis to investigate differences in associations between the study population with imputed data and the complete cases (*n* = 10,323). Thirdly, we executed the abovementioned models per individual SEP indicator, without correction for the other two SEP indicators, to assess the role of the SEP indicators without controlling for the others. All analyses were performed using StataMP 13 (64-bit). To allow for multiple testing, *p*-values< 0.01 were considered to be statistically significant.

## Results

The mean age of the 16,818 participants was 53.4 (SD 12.1) years and 48.5% were female (Table 1). The largest group had finished a secondary vocational education or a work-based learning pathway (27.8%); the mean net household equivalized income was 1563.3 (SD 561.3) euros per month and the mean occupational prestige was 41.7 (SD 13.3). Half of the participants complied with the norm for physical activity; a large number were past smokers, excessive alcohol drinkers, and used a diet of moderate quality. Correlations between the SEP indicators were low to moderate (Pearson’s correlation coefficients between years of education and household equivalized income: 0.34; years of education and occupational prestige: 0.50; and household equivalized income and occupational prestige: 0.34). Overall, the differences in baseline characteristics between the study population (*n* = 16,818) and the excluded participants (*n* = 7650) were small (Supplementary Table 2). Further, the baseline characteristics of the study population (*n* = 16,818) (consisting of only participants with MetS at baseline) and the adult population (*n* = 152,728) of the Lifelines Cohort Study differed only slightly; the study population was older, and included more males and more participants with fewer years of education (Supplementary Table 3).

**Table 1:** Baseline characteristics of the study population

Characteristics	Study population (***n*** = 16,818)^**a**^	Missing values (%)
**Demographic**		
Age (years), mean (SD)	53.4 (12.1)	0
Sex (female)	48.5	0
**Socioeconomic**		
Education (years), mean (SD)	11.3 (2.5)	3.0
Low^b^	44.6	
Middle^b^	32.7	
High^b^	19.7	
Occupational prestige (SIOPS08), mean (SD)	41.7 (13.3)	6.3
Household equivalized income (euros), mean (SD)	1563.3 (561.3)	20.3
**Metabolic syndrome indicators, meeting condition**^**c**^		
Waist circumference^d^	79.1	0
Triglyceride level^e^	76.9	0
HDL cholesterol^f^	69.5	0
Blood pressure^g^	76.6	0
Glucose level^h^	49.3	0.8
**Health behaviors**		
Physical activity^i^	53.5	11.8
Smoking		3.8
Never	35.2	
Past	41.3	
Current	19.7	
Alcohol intake^j^		5.2
None	23.0	
Moderate	35.8	
Excessive	36.0	
Diet quality^k^		15.2
Poor	7.5	
Moderate	69.2	
High	8.2	

*SD* Standard deviation, *SIOPS08* Standard International Occupational Prestige Scale 2008, *HDL* High-density lipoprotein, *LLDS* Lifelines Diet Score; ^a^ % presented, unless indicated otherwise; ^b^ Categories according to Dutch Standard Education Format [34]; ^c^ According to definition of metabolic syndrome by NCEP ATP III; ^d^ ≥ 102 cm in male or ≥ 88 cm in female; ^e^ ≥ 1.70 mmol/l or use of medication for elevated triglycerides; ^f^ <  1.0 mmol/L in male, < 1.3 mmol/L in female or use of lipid-lowering medication; ^g^ Systolic blood pressure ≥ 130 mmHg, diastolic blood pressure ≥ 85 mmHg or use of blood pressure-lowering medication; ^h^ Fasting blood glucose level ≥ 5.6 mmol/l, diagnosis of type 2 diabetes or use of blood glucose-lowering medication; ^i^ Complies with norm of at least 30 min of moderately intensive exercise at least 5 days a week; ^j^ ‘none’ alcohol intake 0 glasses, ‘moderate’ alcohol intake ≤1 glass per day on average, without binge drinking (i.e., >3 glasses on one day for females and >4 glasses on one day for males), or ‘excessive’ alcohol intake >1 glass per day on average, or binge drinking; ^k^ According to Lifelines Diet Score: ‘poor’ diet quality (LLDS 1–16), ‘moderate’ diet quality (LLDS 17–32) or ‘high’ diet quality (LLDS 33–48)

After a median follow-up of 3.8 years, 42.7% of the participants experienced MetS remission. More specifically, MetS is a composite construct consisting of five indicators, and is present if an individual has at least three of these indicators. The majority of the participants (73.8%) had three indicators of MetS at baseline; of these participants 50.5% had two or less indicators upon second assessment, indicating MetS remission (Supplementary Table 4). Individuals with more years of education showed a greater likelihood of MetS remission (OR 1.04, 99% CI: 1.02–1.06) (Table 2, path 1). Individuals with a higher household equivalized income also showed a higher likelihood of MetS remission (OR 1.01, 99% CI: 1.00–1.02). However, when occupational prestige was used as SEP measure, we observed no association with MetS remission.

**Table 2:** Multivariable logistic regression analysis of direct associations between socioeconomic position, health behaviors and metabolic syndrome remission (*n* = 16,818)

	Education	Income	Occupational prestige
	OR (99% CI)	OR (99% CI)	OR (99% CI)
**Path 1. SEP and MetS remission**	1.04 (1.02–1.06)*	1.01 (1.00–1.02)*	1.01 (0.97–1.05)
**Path 2. SEP and health behaviors**			
Physical activity			
No	1.01 (0.98–1.03)	1.00 (0.99–1.01)	1.12 (1.08–1.17)*
Smoking			
Former	0.99 (0.97–1.01)	1.01 (1.00–1.03)*	1.00 (0.96–1.05)
Current	0.94 (0.91–0.97)*	1.00 (0.98–1.01)	0.96 (0.90–1.01)
Alcohol intake			
None	0.93 (0.90–0.96)*	0.97 (0.95–0.98)*	0.94 (0.89–0.99)*
Excessive	0.97 (0.95–1.00)*	1.02 (1.01–1.03)*	0.98 (0.94–1.03)
Diet quality			
Moderate	0.97 (0.93–1.01)	0.98 (0.97–1.00)*	0.95 (0.88–1.03)
Poor	0.88 (0.84–0.93)*	0.98 (0.96–1.00)*	0.91 (0.83–1.00)
**Path 3. Health behavior and MetS remission**			
Physical activity			
No	0.88 (0.80–0.97)*	0.88 (0.80–0.97)*	0.88 (0.80–0.97)*
Smoking			
Former	0.98 (0.89–1.07)	0.98 (0.89–1.07)	0.98 (0.89–1.07)
Current	0.85 (0.76–0.96)*	0.85 (0.76–0.96)*	0.85 (0.76–0.96)*
Alcohol intake			
None	0.87 (0.78–0.97)*	0.87 (0.78–0.97)*	0.87 (0.78–0.97)*
Excessive	1.03 (0.93–1.14)	1.03 (0.93–1.14)	1.03 (0.93–1.14)
Diet quality			
Moderate	0.91 (0.78–1.06)	0.91 (0.78–1.06)	0.91 (0.78–1.06)
Poor	0.73 (0.58–0.91)*	0.73 (0.58–0.91)*	0.73 (0.58–0.91)*
**Path 4. SEP and MetS remission controlled for health behaviors**			
Physical activity	1.04 (1.02–1.06)*	1.01 (1.00–1.02)*	1.01 (0.98–1.05)
Smoking	1.04 (1.02–1.06)*	1.01 (1.00–1.02)*	1.01 (0.97–1.05)
Alcohol intake	1.04 (1.02–1.06)*	1.01 (1.00–1.02)	1.01 (0.97–1.05)
Diet quality	1.04 (1.02–1.06)*	1.01 (1.00–1.02)*	1.01 (0.97–1.05)
Health behaviors combined	1.03 (1.01–1.06)*	1.01 (1.00–1.02)	1.01 (0.97–1.05)

*OR* Odds ratio, *CI* Confidence interval, *SEP* Socioeconomic position, *MetS* Metabolic syndrome; analyses controlled for years of education, household equivalized income, occupational prestige, age and sex at baseline, and time between baseline and second assessment; reference categories for health behaviors: physically active, never smoker, moderate alcohol intake, high diet quality; * *p* < 0.01

**Fig. 2:**
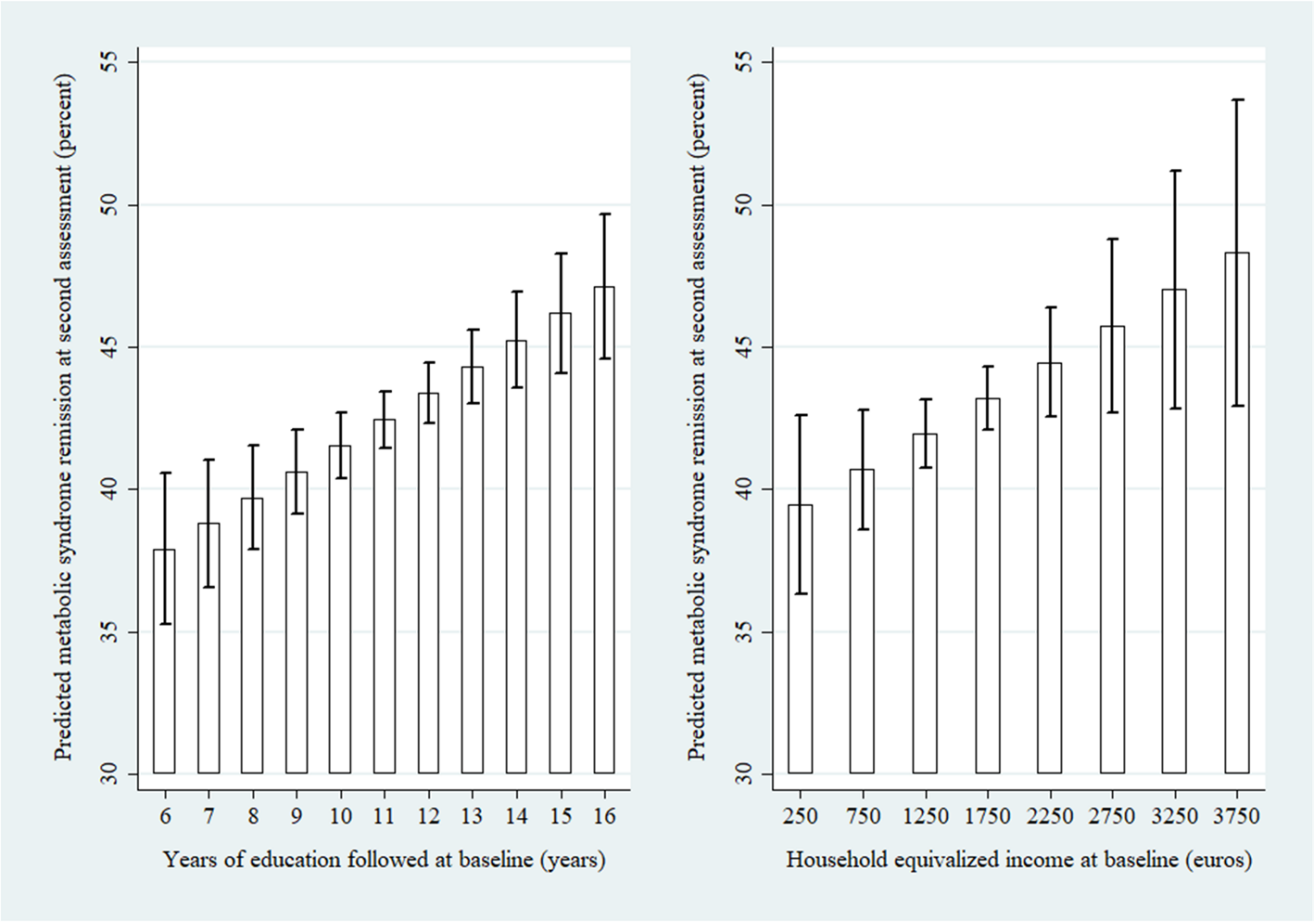
Bar charts of predicted metabolic syndrome remission upon second assessment in percentage (with 99% CI) per year of education followed at baseline (left), and household equivalized income at baseline (right). An average individual in the study population was used (female, 44 years old, follow-up time 3.8 years, educational attainment 12 years, household equivalized income 1600 euros, and occupational prestige 44). In the left figure, years of education were changed; in the right figure, household equivalized income was changed

To illustrate the impact of years of education and household equivalized income on the likelihood of MetS remission, an almost average individual in the study was observed (female, 44 years old, follow-up time 3.8 years, educational attainment 12 years, household equivalized income 1600 euros, and occupational prestige 44); the years of education and the household equivalized income, respectively, were changed (Fig. 2). Regarding education: a participant with 6 years of education had a 38.9% (99% CI: 35.3–40.5%) likelihood of MetS remission, whereas if all parameters in the model were kept the same and only the years of education changed to 16 years, the likelihood of MetS remission increased to 47.1% (99% CI: 44.6–50.0%). Regarding income: a participant with a net household equivalized income of 1250 euros per month, had a 41.9% (99% CI: 40.7–43.2%) likelihood of MetS remission, whereas if all parameters in the model were kept the same and only the household equivalized income changed to 3250 euros per month, the likelihood of MetS remission increased to 47.0% (99% CI: 42.8–51.3%).

Compared to participants with lower occupational prestige, participants with higher occupational prestige were more often total physically inactive (Table 2, path 2). Participants with more years of education were less likely to be current smokers and less likely to have poor diet quality. Similarly, participants with a higher household equivalized income had a higher diet quality. Regardless of the SEP measure used, participants with a higher SEP were less likely to be non-alcohol drinkers. Finally, participants who were not physically active, were current smokers, or had poor diet quality were less likely to experience MetS remission (Table 2, path 3). Interestingly, participants who drank no alcohol were less likely to experience MetS remission.

More highly educated participants had a greater likelihood of remitting MetS; 11.9% of this association was mediated by health behaviors, with diet quality making the greatest contribution (5.3%) (Table 3). Participants with a higher income also had a greater likelihood of remitting MetS, but this association was not mediated by health behaviors. The (in)direct association between occupational prestige and MetS remission was not significant, indicating that health behaviors did not mediate this association.

**Table 3:** Multivariable mediation analysis of health behaviors in associations between socioeconomic position and metabolic syndrome remission using the Karlson-Holm-Breen method (*n* = 16,818)

	Education	Income	Occupational prestige
	**OR (99% CI)**	**OR (99% CI)**	**OR (99% CI)**
**Total association**	1.04 (1.02–1.06)*	1.01 (1.00–1.02)*	1.01 (0.97–1.05)
**Direct association**	1.03 (1.01–1.06)*	1.01 (1.00–1.02)	1.01 (0.97–1.05)
**Indirect association**	1.00 (1.00–1.01)*	1.00 (1.00–1.00)*	1.00 (1.00–1.00)
	**Percentage**	**Percentage**	**Percentage**
**Mediating effects of health behaviors in SEP-MetS remission**			
Physical activity	−0.2	0.5	−37.6
Smoking	3.6	1.4	12.7
Alcohol intake	3.3	11.4	18.4
Diet quality	5.3	2.6	15.0
Health behaviors combined	11.9	15.9	8.5
Change in physical activity	1.1	2.9	−1.4
Change in smoking	−1.3	−1.5	4.3
Change in alcohol intake	−0.8	−2.1	1.7
Change in health behaviors combined	−1.0	−0.6	4.6

*OR* Odds ratio, *CI* Confidence interval, *SEP* Socioeconomic position, *MetS* Metabolic syndrome; analyses controlled for years of education, household equivalized income, occupational prestige, age and sex at baseline, and time between baseline and second assessment; * *p* < 0.01

The additional analyses indicated that a change in health behavior did not explain the associations between SEP and MetS remission (Supplementary Table 5). The results of the sensitivity analyses showed results similar to those of the main analysis (Supplementary Tables 6–13). However, in models assessing the effects of individual SEP indicators on MetS remission, in contrast with the fully adjusted models, occupational prestige did have an effect on MetS remission (Supplementary Tables 14 and 15).

## Discussion

Socioeconomic differences in MetS development are significant [4]. However, less is known about the extent of the role of SEP differences in MetS remission. We showed that both education and income were associated with MetS remission, whereas occupational prestige was not. An average participant with a net household equivalized income of 3250 euros per month has a greater than 12% higher likelihood of MetS remission than a participant with a net household equivalized income of 1250 euros per month. Years of education are an even more important determinant: for the average participant, a total of 16 years of education gives a more than 21% higher likelihood of MetS remission than does a total of 6 years of education. Our results show the importance of conceptualizing SEP as a multidimensional concept, because not only education, but also income has an independent relationship with MetS remission.

As educational differences in MetS remission are greater than income and occupational prestige differences, this study suggests that cultural resources - behavioral values and norms, knowledge, attitudes, beliefs and skills - play an important role in the unequal distribution of MetS remission. Income - the economic position of an individual - seems less important, and occupational prestige - often the operationalization of social standing - has been shown to be no determinant of MetS remission [8–10]. The above is underlined by the sensitivity analysis, in which each SEP indicator was individually modeled on MetS remission. In contrast to the main analysis (with adjustment for income and education), sensitivity analysis indicated that occupational prestige showed an effect on MetS remission (without adjustment for income and education), pointing to education (and probably to a lesser extent, income) as a confounder of the effect of occupational prestige on MetS remission.

Consistent with previous research, we found that individuals with an adverse health status and a lower SEP are disadvantaged, especially when they want to regain good health [5–7]. We also show that a double inequality exists: individuals with a lower SEP not only have a greater likelihood of developing MetS but also a lower likelihood of remitting it [4]. While occupational prestige plays a role in the development of MetS, income plays a role in its remission; however, it is essential for developing and remitting MetS to consider the individual’s educational level.

Our results for health behaviors and MetS remission are mirror images of those observed for MetS development [4, 15, 16]. The lower likelihood of MetS remission among non-drinkers than among moderate drinkers could be related to the fact that we were not able to distinguish different groups among the non-drinkers. The group of non-drinkers includes teetotalers, but also individuals who do not drink because of comorbidities or medication use, resulting in a lower likelihood of MetS remission [35]. Thus heterogeneity in the group of non-drinkers may explain the association with MetS remission.

Major strengths of this study are the longitudinal design and large sample size allowing us to study the relationship between SEP and MetS remission. Further, our results extend previous work, not by explaining SEP differences in MetS development, but focusing on MetS remission to assess SEP differences. In addition, results are likely to be generalizable to the population of the northern part of the Netherlands; it would be interesting to observe if the same results apply to other societal contexts [36]. This study also has some limitations. First, we were not able to distinguish between different groups of non-alcohol drinkers because data about their reasons for non-drinking were not available. A division between these groups would be desirable, because their reasons for not drinking and their chances of MetS remission are based on different grounds. Second, the presence of MetS upon second assessment was determined without including information about medication use at this measurement point (data unavailable). However, the sensitivity analyses, comparing participants with MetS at baseline who did not use prescribed medication upon second assessment with participants who did use prescribed medication upon same measurement point, showed that results were largely the same for both groups. Therefore, we assume that the associations are robust if specific medication use upon second assessment was taken into account.

The results of this study may have important implications for researchers, policy makers and healthcare professionals. Researchers should be aware that when observing socioeconomic health differences, one must not only consider the development of the adverse health outcome, but also its remission. Furthermore, researchers should be aware that SEP differences in health can be affected differently by education, income and occupational prestige; each of these should therefore be considered separately. Interesting for policy makers and healthcare professionals is that interventions and treatment to improve health behaviors in lower educated may only partially (11.9%) reduce educational differences associated with MetS remission, while income and occupational prestige differences on MetS remission will not be changed by improving health behaviors. Future research should focus on other modifiable factors, and the interplay between such factors, that link SEP and MetS remission: health literacy, self-management, perceived difficulties, and living conditions of socioeconomic disadvantaged groups.

## Conclusions

Based on our large-scale longitudinal study we conclude that individuals with a high SEP more often experience MetS remission, compared to their lower SEP counterparts. Given the lack of studies examining the relationship between SEP and MetS remission in a longitudinal design, priority should be given to investigating this relationship in other cohorts to corroborate our findings and to observe which MetS indicator is most important in MetS remission. Second, we found education and income to have different associations with MetS remission, and occupational prestige was not at all associated. Third, individuals with higher education more often experienced MetS remission, mainly because individuals with higher education were more likely to have healthier behaviors. Finally, individuals with a higher income more often experienced MetS remission, regardless of their health behaviors.

## Supplementary Information

**Additional file 1: Table 1**. Measurement in the Lifelines Cohort Study of the variables used in the analyses. **Table 2**. Baseline characteristics of the baseline population (n = 24,458) and a comparison of the study population (n = 16,818) and the participants excluded (n = 7650). **Table 3**. Baseline characteristics of the Lifelines Cohort Study (n = 152,728) and a comparison of the study population (n = 16,818) and the participants excluded (n = 135,910). **Table 4**. Percentages of metabolic syndrome indicators at baseline per percentage of metabolic syndrome indicators upon second assessment for participants with complete data on the five metabolic syndrome indicators. **Table 5**. Multivariable logistic regression analysis of direct associations between socioeconomic position, change in health behaviors and metabolic syndrome remission (n = 16,818). **Table 6**. Multivariable logistic regression analysis of direct associations between socioeconomic position, health behaviors and metabolic syndrome remission among participants without specific medication use taken into account at baseline (n = 13,349). **Table 7**. Multivariable mediation analysis of health behaviors in associations between socioeconomic position and metabolic syndrome remission using the Karlson-Holm-Breen method among participants without specific medication use taken into account at baseline (n = 13,349). **Table 8**. Multivariable logistic regression analysis of direct associations between socioeconomic position, health behaviors and metabolic syndrome remission among participants who have not used general medication during the second assessment (n = 4015). **Table 9**. Multivariable mediation analysis of health behaviors in associations between socioeconomic position and metabolic syndrome remission using the Karlson-Holm-Breen method among participants who have not used general medication during the second assessment (n = 4015). **Table 10**. Multivariable logistic regression analysis of direct associations between socioeconomic position, health behaviors and metabolic syndrome remission among participants who have used general medication during the second assessment (n = 11,172). **Table 11**. Multivariable mediation analysis of health behaviors in associations between socioeconomic position and metabolic syndrome remission using the Karlson-Holm-Breen method among participants who have used general medication during the second assessment (n = 11,172). **Table 12**. Multivariable logistic regression analysis of direct associations between socioeconomic position, health behaviors and metabolic syndrome remission among complete cases (n = 10,323). **Table 13**. Multivariable mediation analysis of health behaviors in associations between socioeconomic position and metabolic syndrome remission using the Karlson-Holm-Breen method among complete cases (n = 10,323). **Table 14**. Multivariable logistic regression analysis of direct associations between each socioeconomic position indicator separately in the model, health behaviors and metabolic syndrome remission (n = 16,818). **Table 15**. Multivariable mediation analysis of health behaviors in associations between each socioeconomic position indicator separately in the model and metabolic syndrome remission using the Karlson-Holm-Breen method (n = 16,818).

## Data Availability

The dataset supporting the conclusions of this article is available from the Lifelines Cohort Study. The generated dataset is not publicly available as it was created and used under license from the Lifelines Cohort Study. Data from the Lifelines Cohort Study are available on request (www.lifelines.nl).
