# Supplementary material for: Understanding socioeconomic differences in metabolic syndrome remission among adults: what is the mediating role of health behaviors?
Source: Int J Behav Nutr Phys Act. 2021 Nov 9;18:147. doi: 10.1186/s12966-021-01217-5 (PMC8577003; doi:10.1186/s12966-021-01217-5)
Supplement: Supplementary file 1 — Additional file 1: Table 1. Measurement in the Lifelines Cohort Study of the variables used in the analyses. Table 2. Baseline characteristics of the baseline population (n = 24,458) and a comparison of the study population (n = 16,818) and the participants excluded (n = 7650). Table 3. Baseline characteristics of the Lifelines Cohort Study (n = 152,728) and a comparison of the study population (n = 16,818) and the participants excluded (n = 135,910). Table 4. Percentages of metabolic syndrome indicators at baseline per percentage of metabolic syndrome indicators upon second assessment for participants with complete data on the five metabolic syndrome indicators. Table 5. Multivariable logistic regression analysis of direct associations between socioeconomic position, change in health behaviors and metabolic syndrome remission (n = 16,818). Table 6. Multivariable logistic regression analysis of direct associations between socioeconomic position, health behaviors and metabolic syndrome remission among participants without specific medication use taken into account at baseline (n = 13,349). Table 7. Multivariable mediation analysis of health behaviors in associations between socioeconomic position and metabolic syndrome remission using the Karlson-Holm-Breen method among participants without specific medication use taken into account at baseline (n = 13,349). Table 8. Multivariable logistic regression analysis of direct associations between socioeconomic position, health behaviors and metabolic syndrome remission among participants who have not used general medication during the second assessment (n = 4015). Table 9. Multivariable mediation analysis of health behaviors in associations between socioeconomic position and metabolic syndrome remission using the Karlson-Holm-Breen method among participants who have not used general medication during the second assessment (n = 4015). Table 10. Multivariable logistic regression analysis of direct associations between socioec [file 12966_2021_1217_MOESM1_ESM.docx]

**SUPPLEMENTARY INFORMATION**

**Table 1. Measurement in the Lifelines Cohort Study of the variables used in the analyses**

| **Variables** | **Measured in the Lifelines Cohort Study** |
| --- | --- |
| Education | ‘What is the highest level of education you have completed?’ Participants had eight answer options, including ‘No education’, ‘Primary education’, ‘Lower or preparatory secondary vocational education’, ‘Junior general secondary education’, ‘Secondary vocational education or work-based learning pathway’, ‘Senior general secondary education, pre-university secondary education’, ‘Higher vocational education’ and ‘University education’. |
| Household equivalized income | Income was measured with the question ‘What is the net income per month. So what you receive in cash and/or on your bank/giro. PLEASE NOTE: if you share the household with someone, the income of your partner(s) must also be included.’. Participants had ten answer options, including ‘Less than 750 euros’, ‘750-1000 euros’, ‘1000-1500 euros’, ‘1500-2000 euros’, ‘2000-2500 euros’, ‘2500-3000 euros’, ‘3000-3500 euros’, ‘more than 3500 euros’, ‘I do not know this.’, ‘I prefer not to answer that.’. |
|  | Household size was measured with the question ‘How many people live from this income?’ ranging from 1 to more than 6 persons. |
| Occupational prestige | ‘If you have or had paid work, what is your last or current profession?’ and ‘Can you explain your profession or function by describing what your main activities are or were?’. Answers were categorized according to the International Standard Classification of Occupations 2008 (ISCO08). |
| Waist circumference | During the physical examination using the SECA 200 measuring tape. |
| Blood pressure | Measurements at baseline consisted of 10 measurements during 10 minutes using the Dinamap PRO 100V2. During the second assessment measurements consisted of three measurements using the Dinamap PRO 100V2. At both occasions, blood pressure was calculated as the average of the last two measurements. |
| Triglyceride | Measured during a laboratory assessment on the day of the fasten blood sample collection. |
| HDL cholesterol | Measured during a laboratory assessment on the day of the fasten blood sample collection. |
| Fasting blood glucose | Measured during a laboratory assessment on the day of the fasten blood sample collection. If participants indicated they did not fast before the blood sample collection, their blood glucose values were assumed not to be valid if they exceeded 5.6 mmol/L according to the cut-off for having MetS and were interpreted as missing value. The determination of type 2 diabetes was based on self-reported questionnaires. |
| Medication use at baseline | The participant was asked about medication use through a questionnaire and by bringing used medications to the research site upon time of the physical examination. All prescribed medications were classified according to the Anatomical Therapeutic Chemical (ATC) coding scheme [23]. |
| Medication use upon second assessment | ‘Do you use prescribed medication?’, participants answering yes or no. Specific information about the prescribed medication was not available upon second assessment. Measurement of these components in the Lifelines Cohort Study was performed in the same way at baseline and the second assessment unless indicated otherwise. |
| Physical activity | Measured using the Short QUestionnaire to Assess Health enhancing physical activity (SQUASH) [24, 25] with questions about occupational, cycling, gardening and sports activities. |
| Smoking | ‘Have you ever smoked for a full year?’ and ‘Do you smoke now, or have you smoked in the past month?’. |
| Alcohol | Measured using the Food Frequency Questionnaire (FFQ) [26, 27] with the questions ‘How often did you drink alcoholic drinks in the past month?’ and ‘How many glasses (i.e. alcoholic drinks) did you drink per day on average?’. |
| Diet | The Lifelines Diet Score (LLDS) is based on the 2015 Dutch Dietary Guidelines [28, 29] and measures the consumption of nine food groups (vegetables, fruit, whole grain products, legumes, nuts, fish, oils- and soft margarines, unsweetened dairy, coffee and tea) with positive and three food groups (red- and processed meat, butter- and hard margarines and sugar-sweetened beverages) with negative health effects. Participants were divided into three groups, according to their LLDS, ‘poor’ diet quality (LLDS 1-16), ‘moderate’ diet quality (LLDS 17-32) or ‘high’ diet quality (LLDS 33-48). |

**Table 2. Baseline characteristics of the baseline population (n = 24,458) and a comparison of the study population (n = 16,818) and the participants excluded (n = 7650)**

| **Characteristic** | **Baseline population (n = 24,458)^a^** | **Study population (n = 16,818)^a,b^** | **Excluded population (n = 7650)^a^** | **Difference between study population and participants excluded^c^** |
| --- | --- | --- | --- | --- |
|  |  |  |  |  |
| **Demographic** |  |  |  |  |
| Age (years), mean (SD) | 52.8 (12.6) | 53.4 (12.1) | 51.4 (13.6) | 1.9 (1.6-2.3) |
| Sex (female) | 49.2 | 48.5 | 50.6 | -2.1 |
|  |  |  |  |  |
| **Socioeconomic** |  |  |  |  |
| Education (years), mean (SD) | 11.2 (2.6) | 11.3 (2.5) | 10.9 (2.6) | 0.3 (0.3-0.4) |
| Low^e^ | 47.2 | 46.0 | 50.0 | -4.0 |
| Middle^e^ | 33.5 | 33.7 | 33.1 | 0.6 |
| High^e^ | 19.2 | 20.3 | 16.9 | 3.4 |
| Occupational prestige (SIOPS08), mean (SD) | 41.1 (13.3) | 41.7 (13.3) | 39.9 (13.2) | 1.8 (1.4-2.2) |
| Household equivalized income (euros), mean (SD) | 1,535.8 (565.2) | 1,563.3 (561.3) | 1,470.3 (569.3) | 92.9 (75.4-110.5) |
|  |  |  |  |  |
| **Metabolic syndrome indicators, meeting condition^e^** |  |  |  |  |
| Waist circumference^f^ | 79.9 | 79.1 | 81.6 | -2.5 |
| Triglyceride level^g^ | 76.8 | 76.9 | 76.5 | 0.4 |
| HDL cholesterol^h^ | 69.8 | 69.5 | 70.6 | -1.1 |
| Blood pressure^i^ | 76.2 | 76.6 | 75.3 | 1.3 |
| Glucose level^j^ | 50.2 | 49.7 | 51.1 | -1.4 |
|  |  |  |  |  |
| **Health behaviors** |  |  |  |  |
| Physical activity^k^ | 58.7 | 60.6 | 54.5 | 6.1 |
| Smoking |  |  |  |  |
| Never | 35.9 | 36.5 | 34.5 | 2.0 |
| Past | 40.8 | 43.0 | 36.1 | 6.9 |
| Current | 23.3 | 20.5 | 29.3 | -8.8 |
| Alcohol intake^l^ |  |  |  |  |
| No | 25.4 | 24.2 | 27.9 | -3.7 |
| Moderate | 36.9 | 37.8 | 35.0 | 2.8 |
| Excessive | 37.7 | 38.0 | 37.1 | 0.9 |
| Diet quality^m^ |  |  |  |  |
| Poor | 10.7 | 9.6 | 13.1 | -3.5 |
| Moderate | 81.1 | 81.6 | 80.0 | 1.6 |
| High | 8.2 | 8.8 | 6.9 | 1.9 |

SD: standard deviation; SIOPS08: Standard International Occupational Prestige Scale 2008; HDL: high-density lipoprotein; LLDS: Lifelines Diet Score; ^a^ % presented, unless indicated otherwise; ^b^ Percentages might differ from Table 1 because missing values are not included and presented in the current table; ^c^ % or mean difference (99% Confidence Interval) are presented; ^d^ Categories according to Dutch Standard Education Format [36]; ^e^ According to definition of metabolic syndrome by NCEP ATP III; ^f^ ≥ 102 cm in male or ≥ 88 cm in female; ^g^ ≥ 1.70 mmol/l or use of medication for elevated triglycerides; ^h^ < 1.0 mmol/L in male, < 1.3 mmol/L in female or use of lipid-lowering medication; ^i^ Systolic blood pressure ≥ 130 mmHg, diastolic blood pressure ≥ 85 mmHg or use of blood pressure-lowering medication; ^j^ Fasting blood glucose level ≥ 5.6 mmol/l, diagnosis of type 2 diabetes or use of blood glucose-lowering medication; ^k^ Complies with norm of at least 30 minutes of moderately intensive exercise at least five days a week; ^l^ ‘no’ alcohol intake 0 glasses, ‘moderate’ alcohol intake ≤1 glass per day on average, without binge drinking (i.e., >3 glasses on one day for females and >4 glasses on one day for males), or ‘excessive’ alcohol intake >1 glass per day on average, or binge drinking; ^m^ According to Lifelines Diet Score: ‘poor’ diet quality (LLDS 1-16), ‘moderate’ diet quality (LLDS 17-32) or ‘high’ diet quality (LLDS 33-48).

**Table 3. Baseline characteristics of the Lifelines Cohort Study (n = 152,728) and a comparison of the study population (n = 16,818) and the participants excluded (n = 135,910)**

| **Characteristic** | **Lifelines Cohort Study population (n = 152,728)^a^** | **Study population (n = 16,818)^a,b^** | **Excluded population (n = 135,910)^a^** | **Difference between study population and participants excluded^c^** |
| --- | --- | --- | --- | --- |
|  |  |  |  |  |
| **Demographic** |  |  |  |  |
| Age (years), mean (SD) | 44.6 (13.1) | 53.4 (12.1) | 43.6 (12.8) | -9.8 (-10.0, -9.6) |
| Sex (female) | 58.5 | 48.5 | 59.7 | 11.2 |
|  |  |  |  |  |
| **Socioeconomic** |  |  |  |  |
| Education (years), mean (SD) | 12.1 (2.5) | 11.3 (2.5) | 12.2 (2.4) | 0.9 (0.9, 1.0) |
| Low^e^ | 30.6 | 46.0 | 28.7 | -17.3 |
| Middle^e^ | 39.5 | 33.7 | 40.3 | 6.6 |
| High^e^ | 29.9 | 20.3 | 31.1 | 10.8 |
| Occupational prestige (SIOPS08), mean (SD) | 43.0 (13.5) | 41.7 (13.3) | 43.2 (13.5) | 1.5 (1.3, 1.7) |
| Household equivalized income (euros), mean (SD) | 1,529.5 (578.7) | 1,563.3 (561.3) | 1,525.5 (580.6) | -37.8 (-48.2, -27.4) |
|  |  |  |  |  |
| **Metabolic syndrome indicators, meeting condition^e^** |  |  |  |  |
| Waist circumference^f^ | 35.1 | 79.1 | 29.7 | -49.4 |
| Triglyceride level^g^ | 20.2 | 76.9 | 12.9 | -64.0 |
| HDL cholesterol^h^ | 19.8 | 69.5 | 13.4 | -56.1 |
| Blood pressure^i^ | 37.1 | 76.6 | 32.2 | -44.4 |
| Glucose level^j^ | 13.1 | 49.7 | 8.3 | -41.4 |
|  |  |  |  |  |
| **Health behaviors** |  |  |  |  |
| Physical activity^k^ | 54.5 | 60.6 | 53.8 | -6.8 |
| Smoking |  |  |  |  |
| Never smoker | 46.2 | 36.5 | 47.4 | 10.9 |
| Past smoker | 31.9 | 43.0 | 30.4 | -12.6 |
| Current smoker | 22.0 | 20.5 | 22.2 | 1.7 |
| Alcohol intake^l^ |  |  |  |  |
| No alcohol intake | 20.3 | 24.2 | 19.8 | -4.4 |
| Moderate alcohol intake | 43.1 | 37.8 | 43.7 | 5.9 |
| Excessive alcohol intake | 36.6 | 38.0 | 36.4 | -1.6 |
| Diet quality^m^ |  |  |  |  |
| Poor | 11.0 | 9.6 | 11.1 | 1.5 |
| Moderate | 80.6 | 81.6 | 80.6 | -1.0 |
| High | 8.5 | 8.8 | 8.5 | -0.3 |

SD: standard deviation; SIOPS08: Standard International Occupational Prestige Scale 2008; HDL: high-density lipoprotein; LLDS: Lifelines Diet Score; ^a^ % presented, unless indicated otherwise; ^b^ Percentages might differ from Table 1 because missing values are not included and presented in the current table; ^c^ % or mean difference (99% Confidence Interval) are presented; ^d^ Categories according to Dutch Standard Education Format [36]; ^e^ According to definition for metabolic syndrome by NCEP ATP III; ^f^ ≥ 102 cm in male or ≥ 88 cm in female; ^g^ ≥ 1.70 mmol/l or use of medication for elevated triglycerides; ^h^ < 1.0 mmol/L in male, < 1.3 mmol/L in female or use of lipid-lowering medication; ^i^ Systolic blood pressure ≥ 130 mmHg, diastolic blood pressure ≥ 85 mmHg or use of blood pressure-lowering medication; ^j^ Fasting blood glucose level ≥ 5.6 mmol/l, diagnosis of type 2 diabetes or use of blood glucose-lowering medication; ^k^ Complies with norm of at least 30 minutes of moderately intensive exercise at least five days a week; ^l^ ‘no’ alcohol intake 0 glasses, ‘moderate’ alcohol intake ≤1 glass per day on average, without binge drinking (i.e., >3 glasses on one day for females and >4 glasses on one day for males), or ‘excessive’ alcohol intake >1 glass per day on average, or binge drinking; ^m^ According to Lifelines Diet Score: ‘poor’ diet quality (LLDS 1-16), ‘moderate’ diet quality (LLDS 17-32) or ‘high’ diet quality (LLDS 33-48).

**Table 4. Percentages of metabolic syndrome indicators at baseline per percentage of metabolic syndrome indicators upon second assessment for participants with complete data on the five metabolic syndrome indicators**

|  | **Metabolic syndrome indicators at baseline** | | |
| --- | --- | --- | --- |
|  | **3** | **4** | **5** |
| **Metabolic syndrome indicators upon second assessment** |  |  |  |
| **0** | 2.8 (3.8) | 2.7 (1.7) | 0.0 (0.0) |
| **1** | 11.8 (16.0) | 12.2 (6.8) | 0.1 (2.5) |
| **2** | 22.7 (30.7) | 10.8 (20.3) | 0.6 (15.0) |
| **3** | 25.2 (34.1) | 1.6 (28.9) | 0.8 (20.6) |
| **4** | 9.7 (13.2) | 1.8 (33.3) | 1.3 (31.9) |
| **5** | 1.6 (2.2) | 0.5 (9.1) | 1.2 (30.0) |
| **Total** | 73.8 (100) | 22.2 (100) | 4.1 (100) |

Shown are the percentages that add up to the total percentage per number of MetS indicators at baseline. The percentages that add up to 100% are shown in parentheses.

The percentages for two or fewer MetS indicators on the second assessment do not add up to 42.7% (the percentage of participants who remit from MetS in our study) because we only used the complete cases in the current calculation. The MetS variables in our study could also be based on information from three or four indicators. As stated in the method: "Participants were excluded if three or more MetS indicators were missing or if it was not possible to determine whether or not they had MetS when they had provided information on three or four indicators only."

**Table 5. Multivariable logistic regression analysis of direct associations between socioeconomic position, change in health behaviors and metabolic syndrome remission (n = 16,818)**

|  | **Education** | **Income** | **Occupational prestige** |
| --- | --- | --- | --- |
|  | **OR (99% CI)** | **OR (99% CI)** | **OR (99% CI)** |
| **Path 1. SEP and MetS remission** | 1.04 (1.02-1.06)* | 1.01 (1.00-1.02)* | 1.01 (0.98-1.05) |
| **Path 2. SEP and change in health behavior** |  |  |  |
| Physical activity |  |  |  |
| No | 0.98 (0.95-1.01) | 1.00 (0.98-1.01) | 0.99 (0.94-1.05) |
| Decreased | 0.98 (0.94-1.02) | 0.99 (0.97-1.01) | 1.01 (0.93-1.09) |
| Smoking |  |  |  |
| No | 1.01 (0.97-1.04) | 0.99 (0.98-1.01) | 0.97 (0.90-1.04) |
| Increased | 0.98 (0.91-1.05) | 0.99 (0.95-1.02) | 0.96 (0.83-1.10) |
| Alcohol intake |  |  |  |
| No | 1.03 (0.99-1.06) | 1.02 (1.00-1.03)* | 0.99 (0.93-1.05) |
| Increased | 1.02 (0.97-1.07) | 1.03 (1.01-1.05)* | 0.98 (0.90-1.06) |
| **Path 3. Change in health behavior and MetS remission** |  |  |  |
| Physical activity |  |  |  |
| No | 0.85 (0.75-0.97)* | 0.85 (0.75-0.97)* | 0.85 (0.75-0.97)* |
| Decreased | 0.75 (0.64-0.89)* | 0.75 (0.64-0.89)* | 0.75 (0.64-0.89)* |
| Smoking |  |  |  |
| No | 1.09 (0.95-1.26) | 1.09 (0.95-1.26) | 1.09 (0.95-1.26) |
| Increased | 1.37 (1.02-1.85)* | 1.37 (1.02-1.85)* | 1.37 (1.02-1.85)* |
| Alcohol intake |  |  |  |
| No | 0.88 (0.77-1.02) | 0.88 (0.77-1.02) | 0.88 (0.77-1.02) |
| Increased | 0.94 (0.77-1.13) | 0.94 (0.77-1.13) | 0.94 (0.77-1.13) |
| **Path 4. SEP and MetS remission controlled for health behaviors at baseline and change in health behaviors** |  |  |  |
| Physical activity | 1.04 (1.02-1.06)* | 1.01 (1.00-1.02)* | 1.02 (0.98-1.06) |
| Smoking | 1.04 (1.02-1.06)* | 1.01 (1.00-1.02)* | 1.01 (0.97-1.05) |
| Alcohol intake | 1.04 (1.02-1.06)* | 1.01 (1.00-1.02)* | 1.01 (0.97-1.05) |
| Health behaviors combined | 1.03 (1.01-1.06)* | 1.01 (1.00-1.02) | 1.01 (0.97-1.05) |
|  | **Percentage** | **Percentage** | **Percentage** |
| **Mediating effects SEP and MetS remission** |  |  |  |
| Change in physical activity | 1.3* | 2.9 | -1.4 |
| Change in smoking | -0.9* | -1.5 | 4.3 |
| Change in alcohol intake | -2.3* | -2.1 | 1.7 |
| Change in health behaviors combined | -1.1* | -0.6 | 4.6 |
| OR: odds ratio; CI: confidence interval; SEP: socioeconomic position; MetS: metabolic syndrome; analyses controlled for years of education, household equivalized income, occupational prestige, age and sex at baseline and time between baseline and second assessment; no change in health behavior was the reference category; * p<0.01. | | | |

**Table 6. Multivariable logistic regression analysis of direct associations between socioeconomic position, health behaviors and metabolic syndrome remission among participants without specific medication use taken into account at baseline (n = 13,349)**

|  | **Education** | **Income** | **Occupational prestige** |
| --- | --- | --- | --- |
|  | **OR (99% CI)** | **OR (99% CI)** | **OR (99% CI)** |
| **Path 1. SEP and MetS remission** | 1.03 (1.01-1.06)* | 1.01 (1.00-1.02)* | 1.01 (0.96-1.05) |
| **Path 2. SEP and health behavior** |  |  |  |
| Physical activity |  |  |  |
| No | 1.02 (0.99-1.05) | 1.00 (0.99-1.01) | 1.12 (1.06-1.18)* |
| Smoking |  |  |  |
| Former | 0.99 (0.97-1.02) | 1.01 (1.00-1.03) | 0.99 (0.94-1.05) |
| Current | 0.94 (0.91-0.97)* | 1.00 (0.99-1.02) | 0.95 (0.90-1.01) |
| Alcohol intake |  |  |  |
| None | 0.92 (0.89-0.95)* | 0.97 (0.96-0.98)* | 0.94 (0.89-0.99)* |
| Excessive | 0.97 (0.94-0.99)* | 1.02 (1.01-1.03)* | 0.96 (0.91-1.02) |
| Diet quality |  |  |  |
| Moderate | 0.98 (0.93-1.02) | 0.98 (0.96-1.00)* | 0.94 (0.86-1.02) |
| Poor | 0.89 (0.84-0.94)* | 0.98 (0.95-1.01) | 0.89 (0.81-0.99) |
| **Path 3. Health behavior and MetS remission** |  |  |  |
| Physical activity |  |  |  |
| No | 0.93 (0.83-1.03) | 0.93 (0.83-1.03) | 0.93 (0.83-1.03) |
| Smoking |  |  |  |
| Former | 0.96 (0.85-1.08) | 0.96 (0.85-1.08) | 0.96 (0.85-1.08) |
| Current | 0.85 (0.75-0.98)* | 0.85 (0.75-0.98)* | 0.85 (0.75-0.98)* |
| Alcohol intake |  |  |  |
| None | 0.85 (0.74-0.96)* | 0.85 (0.74-0.96)* | 0.85 (0.74-0.96)* |
| Excessive | 1.06 (0.94-1.20) | 1.06 (0.94-1.20) | 1.06 (0.94-1.20) |
| Diet quality |  |  |  |
| Moderate | 0.92 (0.75-1.13) | 0.92 (0.75-1.13) | 0.92 (0.75-1.13) |
| Poor | 0.80 (0.62-1.03) | 0.80 (0.62-1.03) | 0.80 (0.62-1.03) |
| **Path 4. SEP and MetS remission controlled for health behaviors** |  |  |  |
| Physical activity | 1.03 (1.01-1.06)* | 1.01 (1.00-1.02)* | 1.01 (0.97-1.06) |
| Smoking | 1.03 (1.01-1.06)* | 1.01 (1.00-1.02)* | 1.01 (0.96-1.05) |
| Alcohol intake | 1.03 (1.01-1.06)* | 1.01 (1.00-1.02)* | 1.01 (0.96-1.05) |
| Diet quality | 1.03 (1.01-1.06)* | 1.01 (1.00-1.02)* | 1.01 (0.96-1.05) |
| Health behaviors combined | 1.03 (1.00-1.05)* | 1.01 (1.00-1.02)* | 1.01 (0.96-1.05) |
| OR: odds ratio; CI: confidence interval; SEP: socioeconomic position; MetS: metabolic syndrome; analyses controlled for years of education, household equivalized income, occupational prestige, age and sex at baseline, and time between baseline and second assessment; reference categories for health behaviors: physically active, never smoker, moderate alcohol intake, high diet quality; * p<0.01. | | | |

**Table 7. Multivariable mediation analysis of health behaviors in associations between socioeconomic position and metabolic syndrome remission using the Karlson-Holm-Breen method among participants without specific medication use taken into account at baseline (n = 13,349)**

|  | **Education** | **Income** | **Occupational prestige** |
| --- | --- | --- | --- |
|  | **OR (99% CI)** | **OR (99% CI)** | **OR (99% CI)** |
| **Total association** | 1.03 (1.01-1.06)* | 1.01 (1.00-1.02)* | 1.01 (0.96-1.05) |
| **Direct association** | 1.03 (1.00-1.05)* | 1.01 (1.00-1.02)* | 1.01 (0.96-1.05) |
| **Indirect association** | 1.00 (1.00-1.01)* | 1.00 (1.00-1.00)* | 1.00 (1.00-0.99) |
|  |  |  |  |
|  | **Percentage** | **Percentage** | **Percentage** |
| **Mediating effects SEP and MetS remission** |  |  |  |
| Physical activity | -1.0 | 0.0 | -33.5 |
| Smoking | 4.5 | -0.3 | 17.7 |
| Alcohol intake | 5.1 | 11.6 | 21.5 |
| Diet quality | 4.8 | 1.4 | 17.6 |
| Health behaviors combined | 13.4 | 12.8 | 23.3 |
| OR: odds ratio; CI: confidence interval; SEP: socioeconomic position; MetS: metabolic syndrome; analyses controlled for years of education, household equivalized income, occupational prestige, age and sex at baseline, and time between baseline and second assessment; * p<0.01. | | | |

**Table 8. Multivariable logistic regression analysis of direct associations between socioeconomic position, health behaviors and metabolic syndrome remission among participants who have not used general medication during the second assessment (n = 4015)**

|  | **Education** | **Income** | **Occupational prestige** |
| --- | --- | --- | --- |
|  | **OR (99% CI)** | **OR (99% CI)** | **OR (99% CI)** |
| **Path 1. SEP and MetS remission** | 1.01 (0.97-1.05) | 1.00 (0.99-1.02) | 1.06 (0.98-1.15) |
| **Path 2. SEP and health behavior** |  |  |  |
| Physical activity |  |  |  |
| No | 1.02 (0.99-1.05) | 1.00 (0.99-1.01) | 1.12 (1.06-1.18)* |
| Smoking |  |  |  |
| Former | 0.99 (0.97-1.02) | 1.01 (1.00-1.03) | 0.99 (0.94-1.05) |
| Current | 0.94 (0.91-0.97)* | 1.00 (0.99-1.02) | 0.95 (0.90-1.01) |
| Alcohol intake |  |  |  |
| None | 0.92 (0.89-0.95)* | 0.97 (0.96-0.98)* | 0.94 (0.89-0.99)* |
| Excessive | 0.97 (0.94-0.99)* | 1.02 (1.01-1.03)* | 0.96 (0.91-1.02) |
| Diet quality |  |  |  |
| Moderate | 0.98 (0.93-1.02) | 0.98 (0.96-1.00) | 0.94 (0.86-1.02) |
| Poor | 0.89 (0.84-0.94)* | 0.98 (0.95-1.01) | 0.89 (0.81-0.99)* |
| **Path 3. Health behavior and MetS remission** |  |  |  |
| Physical activity |  |  |  |
| No | 0.93 (0.83-1.03) | 0.93 (0.83-1.03) | 0.93 (0.83-1.03) |
| Smoking |  |  |  |
| Former | 0.96 (0.85-1.08) | 0.96 (0.85-1.08) | 0.96 (0.85-1.08) |
| Current | 0.85 (0.75-0.98)* | 0.85 (0.75-0.98)* | 0.85 (0.75-0.98)* |
| Alcohol intake |  |  |  |
| None | 0.85 (0.74-0.96)* | 0.85 (0.74-0.96)* | 0.85 (0.74-0.96)* |
| Excessive | 1.06 (0.94-1.20) | 1.06 (0.94-1.20) | 1.06 (0.94-1.20) |
| Diet quality |  |  |  |
| Moderate | 0.92 (0.75-1.13) | 0.92 (0.75-1.13) | 0.92 (0.75-1.13) |
| Poor | 0.80 (0.62-1.03) | 0.80 (0.62-1.03) | 0.80 (0.62-1.03) |
| **Path 4. SEP and MetS remission controlled for health behaviors** |  |  |  |
| Physical activity | 1.03 (1.01-1.06)* | 1.01 (1.00-1.02)* | 1.01 (0.97-1.06) |
| Smoking | 1.03 (1.01-1.06)* | 1.01 (1.00-1.02)* | 1.01 (0.96-1.05) |
| Alcohol intake | 1.03 (1.01-1.06)* | 1.01 (1.00-1.02)* | 1.01 (0.96-1.05) |
| Diet quality | 1.03 (1.01-1.06)* | 1.01 (1.00-1.02)* | 1.01 (0.96-1.05) |
| Health behaviors combined | 1.03 (1.00-1.05)* | 1.01 (1.00-1.02)* | 1.01 (0.96-1.05) |
| OR: odds ratio; CI: confidence interval; SEP: socioeconomic position; MetS: metabolic syndrome; analyses controlled for years of education, household equivalized income, occupational prestige, age and sex at baseline, and time between baseline and second assessment; reference categories for health behaviors: physically active, never smoker, moderate alcohol intake, high diet quality; * p<0.01. | | | |

**Table 9. Multivariable mediation analysis of health behaviors in associations between socioeconomic position and metabolic syndrome remission using the Karlson-Holm-Breen method among participants who have not used general medication during the second assessment (n = 4015)**

|  | **Education** | **Income** | **Occupational prestige** |
| --- | --- | --- | --- |
|  | **OR (99% CI)** | **OR (99% CI)** | **OR (99% CI)** |
| **Total association** | 1.01 (0.97-1.05) | 1.00 (0.99-1.02) | 1.06 (0.98-1.15) |
| **Direct association** | 1.01 (0.97-1.05) | 1.00 (0.99-1.02) | 1.06 (0.98-1.15) |
| **Indirect association** | 1.00 (1.00-1.01) | 1.00 (1.00-1.00) | 1.00 (0.99-1.01) |
|  |  |  |  |
|  | **Percentage** | **Percentage** | **Percentage** |
| **Mediating effects SEP and MetS remission** |  |  |  |
| Physical activity | -12.8 | -1.1 | -5.4 |
| Smoking | 18.1 | 0.3 | 1.0 |
| Alcohol intake | 1.1 | 0.3 | 1.6 |
| Diet quality | 31.2 | -10.2 | 4.0 |
| Health behaviors combined | 37.6 | -10.7 | 1.2 |
| OR: odds ratio; CI: confidence interval; SEP: socioeconomic position; MetS: metabolic syndrome; analyses controlled for years of education, household equivalized income, occupational prestige, age and sex at baseline, and time between baseline and second assessment; * p<0.01. | | | |

**Table 10. Multivariable logistic regression analysis of direct associations between socioeconomic position, health behaviors and metabolic syndrome remission among participants who have used general medication during the second assessment (n = 11,172)**

|  | **Education** | **Income** | **Occupational prestige** |
| --- | --- | --- | --- |
|  | **OR (99% CI)** | **OR (99% CI)** | **OR (99% CI)** |
| **Path 1. SEP and MetS remission** | 1.05 (1.02-1.08)* | 1.01 (1.00-1.02) | 1.00 (0.95-1.04) |
| **Path 2. SEP and health behavior** |  |  |  |
| Physical activity |  |  |  |
| No | 0.98 (0.95-1.02) | 1.00 (0.99-1.01) | 1.11 (1.04-1.18)* |
| Smoking |  |  |  |
| Former | 1.00 (0.97-1.03) | 1.02 (1.00-1.03)* | 1.00 (0.94-1.06) |
| Current | 0.94 (0.91-0.98)* | 1.00 (0.98-1.01) | 0.94 (0.88-1.02) |
| Alcohol intake |  |  |  |
| None | 0.92 (0.89-0.95)* | 0.97 (0.95-0.99)* | 0.94 (0.89-1.00) |
| Excessive | 0.97 (0.94-1.00) | 1.02 (1.01-1.04)* | 0.99 (0.94-1.05) |
| Diet quality |  |  |  |
| Moderate | 0.97 (0.92-1.01) | 0.99 (0.97-1.01) | 0.95 (0.88-1.04) |
| Poor | 0.90 (0.84-0.96)* | 0.99 (0.96-1.02) | 0.90 (0.79-1.02) |
| **Path 3. Health behavior and MetS remission** |  |  |  |
| Physical activity |  |  |  |
| No | 0.89 (0.79-1.02) | 0.89 (0.79-1.02) | 0.89 (0.79-1.02) |
| Smoking |  |  |  |
| Former | 0.94 (0.83-1.06) | 0.94 (0.83-1.06) | 0.94 (0.83-1.06) |
| Current | 0.84 (0.73-0.98)* | 0.84 (0.73-0.98)* | 0.84 (0.73-0.98)* |
| Alcohol intake |  |  |  |
| None | 0.85 (0.74-0.97)* | 0.85 (0.74-0.97)* | 0.85 (0.74-0.97)* |
| Excessive | 1.00 (0.88-1.13) | 1.00 (0.88-1.13) | 1.00 (0.88-1.13) |
| Diet quality |  |  |  |
| Moderate | 0.91 (0.75-1.11) | 0.91 (0.75-1.11) | 0.91 (0.75-1.11) |
| Poor | 0.74 (0.58-0.96)* | 0.74 (0.58-0.96)* | 0.74 (0.58-0.96)* |
| **Path 4. SEP and MetS remission controlled for health behaviors** |  |  |  |
| Physical activity | 1.05 (1.02-1.08)* | 1.01 (1.00-1.02) | 1.00 (0.95-1.05) |
| Smoking | 1.05 (1.02-1.07)* | 1.01 (1.00-1.02) | 0.99 (0.95-1.04) |
| Alcohol intake | 1.05 (1.02-1.07)* | 1.01 (1.00-1.02) | 0.99 (0.95-1.04) |
| Diet quality | 1.05 (1.02-1.07)* | 1.01 (1.00-1.02) | 0.99 (0.95-1.04) |
| Health behaviors combined | 1.04 (1.02-1.07)* | 1.01 (1.00-1.02) | 0.99 (0.95-1.04) |
| OR: odds ratio; CI: confidence interval; SEP: socioeconomic position; MetS: metabolic syndrome; analyses controlled for years of education, household equivalized income, occupational prestige, age and sex at baseline, and time between baseline and second assessment; reference categories for health behaviors: physically active, never smoker, moderate alcohol intake, high diet quality; * p<0.01. | | | |

**Table 11. Multivariable mediation analysis of health behaviors in associations between socioeconomic position and metabolic syndrome remission using the Karlson-Holm-Breen method among participants who have used general medication during the second assessment (n = 11,172)**

|  | **Education** | **Income** | **Occupational prestige** |
| --- | --- | --- | --- |
|  | **OR (99% CI)** | **OR (99% CI)** | **OR (99% CI)** |
| **Total association** | 1.05 (1.02-1.08)* | 1.01 (1.00-1.02) | 1.00 (0.95-1.04) |
| **Direct association** | 1.04 (1.02-1.07)* | 1.01 (1.00-1.02) | 0.99 (0.95-1.04) |
| **Indirect association** | 1.00 (1.00-1.01)* | 1.00 (1.00-1.00)* | 1.00 (1.00-1.01) |
|  |  |  |  |
|  | **Percentage** | **Percentage** | **Percentage** |
| **Mediating effects SEP and MetS remission** |  |  |  |
| Physical activity | 0.8 | 0.3 | 20.5 |
| Smoking | 2.3 | 0.4 | -8.8 |
| Alcohol intake | 3.4 | 11.3 | -16.4 |
| Diet quality | 3.3 | 0.8 | -6.7 |
| Health behaviors combined | 9.8 | 12.8 | -11.3 |
| OR: odds ratio; CI: confidence interval; SEP: socioeconomic position; MetS: metabolic syndrome; analyses controlled for years of education, household equivalized income, occupational prestige, age and sex at baseline, and time between baseline and second assessment; * p<0.01. | | | |

**Table 12. Multivariable logistic regression analysis of direct associations between socioeconomic position, health behaviors and metabolic syndrome remission among complete cases (n = 10,323)**

|  | **Education** | **Income** | **Occupational prestige** |
| --- | --- | --- | --- |
|  | **OR (99% CI)** | **OR (99% CI)** | **OR (99% CI)** |
| **Path 1. SEP and MetS remission** | 1.04 (1.02-1.07)* | 1.01 (1.00-1.02)* | 1.00 (0.95-1.05) |
| **Path 2. SEP and health behavior** |  |  |  |
| Physical activity |  |  |  |
| No | 1.01 (0.98-1.04) | 1.00 (0.99-1.01) | 1.11 (1.06-1.17)* |
| Smoking |  |  |  |
| Former | 0.98 (0.95-1.01) | 1.01 (1.00-1.02)* | 1.01 (0.96-1.06) |
| Current | 0.93 (0.90-0.97)* | 1.00 (0.99-1.02) | 0.97 (0.91-1.04) |
| Alcohol intake |  |  |  |
| None | 0.93 (0.90-0.96)* | 0.97 (0.96-0.99)* | 0.92 (0.86-0.98)* |
| Excessive | 0.98 (0.95-1.01) | 1.03 (1.01-1.04)* | 0.97 (0.92-1.02) |
| Diet quality |  |  |  |
| Moderate | 0.96 (0.92-1.01) | 0.98 (0.97-1.00) | 0.94 (0.86-1.02) |
| Poor | 0.89 (0.84-0.95)* | 0.98 (0.95-1.00) | 0.88 (0.79-0.98)* |
| **Path 3. Health behavior and MetS remission** |  |  |  |
| Physical activity |  |  |  |
| No | 0.88 (0.78-0.98)* | 0.88 (0.78-0.98)* | 0.88 (0.78-0.98)* |
| Smoking |  |  |  |
| Former | 0.97 (0.86-1.09) | 0.97 (0.86-1.09) | 0.97 (0.86-1.09) |
| Current | 0.88 (0.76-1.03) | 0.88 (0.76-1.03) | 0.88 (0.76-1.03) |
| Alcohol intake |  |  |  |
| None | 0.83 (0.72-0.96)* | 0.83 (0.72-0.96)* | 0.83 (0.72-0.96)* |
| Excessive | 1.03 (0.92-1.17) | 1.03 (0.92-1.17) | 1.03 (0.92-1.17) |
| Diet quality |  |  |  |
| Moderate | 0.86 (0.71-1.04) | 0.86 (0.71-1.04) | 0.86 (0.71-1.04) |
| Poor | 0.69 (0.53-0.88)* | 0.69 (0.53-0.88)* | 0.69 (0.53-0.88)* |
| **Path 4. SEP and MetS remission controlled for health behaviors** |  |  |  |
| Physical activity | 1.04 (1.02-1.07)* | 1.01 (1.00-1.02)* | 1.00 (0.96-1.05) |
| Smoking | 1.04 (1.02-1.07)* | 1.01 (1.00-1.02)* | 1.00 (0.95-1.05) |
| Alcohol intake | 1.04 (1.02-1.07)* | 1.01 (1.00-1.02)* | 1.00 (0.95-1.04) |
| Diet quality | 1.04 (1.02-1.07)* | 1.01 (1.00-1.02)* | 1.00 (0.95-1.05) |
| Health behaviors combined | 1.04 (1.01-1.07)* | 1.01 (1.00-1.02) | 1.00 (0.95-1.05) |
| OR: odds ratio; CI: confidence interval; SEP: socioeconomic position; MetS: metabolic syndrome; analyses controlled for years of education, household equivalized income, occupational prestige, age and sex at baseline, and time between baseline and second assessment; reference categories for health behaviors: physically active, never smoker, moderate alcohol intake, high diet quality; * p<0.01. | | | |

**Table 13. Multivariable mediation analysis of health behaviors in associations between socioeconomic position and metabolic syndrome remission using the Karlson-Holm-Breen method among complete cases (n = 10,323)**

|  | **Education** | **Income** | **Occupational prestige** |
| --- | --- | --- | --- |
|  | **OR (99% CI)** | **OR (99% CI)** | **OR (99% CI)** |
| **Total association** | 1.04 (1.02-1.07)* | 1.01 (1.00-1.02)* | 1.00 (0.97-1.04) |
| **Direct association** | 1.04 (1.02-1.06)* | 1.01 (1.00-1.02) | 1.00 (0.96-1.03) |
| **Indirect association** | 1.00 (1.00-1.01)* | 1.00 (1.00-1.00)* | 1.00 (1.00-1.01) |
|  |  |  |  |
|  | **Percentage** | **Percentage** | **Percentage** |
| **Mediating effects SEP and MetS remission** |  |  |  |
| Physical activity | -0.4 | 0.6 | 1691.8 |
| Smoking | 2.3 | 0.0 | -258.0 |
| Alcohol intake | 3.4 | 11.0 | -1225.1 |
| Diet quality | 4.5 | 2.9 | -1137.4 |
| Health behaviors combined | 9.8 | 14.6 | -928.6 |
| OR: odds ratio; CI: confidence interval; SEP: socioeconomic position; MetS: metabolic syndrome; analyses controlled for years of education, household equivalized income, occupational prestige, age and sex at baseline, and time between baseline and second assessment; * p<0.01. | | | |

**Table 14. Multivariable logistic regression analysis of direct associations between each socioeconomic position indicator separately in the model, health behaviors and metabolic syndrome remission (n = 16,818)**

|  | **Education** | **Income** | **Occupational prestige** |
| --- | --- | --- | --- |
|  | **OR (99% CI)** | **OR (99% CI)** | **OR (99% CI)** |
| **Path 1. SEP and MetS remission** | 1.05 (1.03-1.07)* | 1.02 (1.01-1.03)* | 1.06 (1.03-1.09)* |
| **Path 2. SEP and health behaviors** |  |  |  |
| Physical activity |  |  |  |
| No | 1.04 (1.02-1.06)* | 1.01 (1.00-1.02) | 1.13 (1.09-1.17)* |
| Smoking |  |  |  |
| Former | 1.00 (0.99-1.02) | 1.01 (1.00-1.02)* | 1.02 (0.98-1.05) |
| Current | 0.93 (0.91-0.95)* | 0.98 (0.97-1.00)* | 0.90 (0.86-0.94)* |
| Alcohol intake |  |  |  |
| None | 0.89 (0.87-0.91)* | 0.95 (0.94-0.96)* | 0.84 (0.81-0.88)* |
| Excessive | 0.99 (0.97-1.01) | 1.01 (1.00-1.03)* | 0.99 (0.95-1.03) |
| Diet quality |  |  |  |
| Moderate | 0.94 (0.91-0.97)* | 0.97 (0.96-0.99)* | 0.90 (0.84-0.96)* |
| Poor | 0.85 (0.81-0.88)* | 0.95 (0.93-0.97)* | 0.79 (0.73-0.85)* |
| **Path 3. Health behavior and MetS remission** |  |  |  |
| Physical activity |  |  |  |
| No | 0.89 (0.81-0.97)* | 0.89 (0.81-0.98)* | 0.89 (0.81-0.97)* |
| Smoking |  |  |  |
| Former | 0.98 (0.89-1.08) | 0.97 (0.89-1.07) | 0.98 (0.89-1.07) |
| Current | 0.85 (0.76-0.96)* | 0.84 (0.75-0.95)* | 0.84 (0.75-0.95)* |
| Alcohol intake |  |  |  |
| None | 0.86 (0.77-0.96)* | 0.86 (0.77-0.96)* | 0.85 (0.76-0.95)* |
| Excessive | 1.03 (0.93-1.14) | 1.02 (0.93-1.13) | 1.03 (0.93-1.14) |
| Diet quality |  |  |  |
| Moderate | 0.90 (0.77-1.06) | 0.90 (0.77-1.05) | 0.90 (0.77-1.05) |
| Poor | 0.73 (0.58-0.91)* | 0.71 (0.57-0.89)* | 0.71 (0.57-0.89)* |
| **Path 4. SEP and MetS remission controlled for health behaviors** |  |  |  |
| Physical activity | 1.05 (1.03-1.07)* | 1.02 (1.01-1.03)* | 1.06 (1.03-1.10)* |
| Smoking | 1.05 (1.03-1.07)* | 1.02 (1.01-1.03)* | 1.06 (1.03-1.09)* |
| Alcohol intake | 1.05 (1.03-1.06)* | 1.02 (1.01-1.03)* | 1.06 (1.02-1.09)* |
| Diet quality | 1.05 (1.03-1.06)* | 1.02 (1.01-1.03)* | 1.06 (1.02-1.09)* |
| Health behaviors combined | 1.04 (1.03-1.06)* | 1.01 (1.01-1.02)* | 1.05 (1.02-1.09)* |

OR: odds ratio; CI: confidence interval; SEP: socioeconomic position; MetS: metabolic syndrome; analyses controlled for years of education, household equivalized income, occupational prestige, age and sex at baseline, and time between baseline and second assessment; reference categories for health behaviors: physically active, never smoker, moderate alcohol intake, high diet quality; * p<0.01.

**Table 15. Multivariable mediation analysis of health behaviors in associations between each socioeconomic position indicator separately in the model and metabolic syndrome remission using the Karlson-Holm-Breen method (n = 16,818)**

|  | **Education** | **Income** | **Occupational prestige** |
| --- | --- | --- | --- |
|  | **OR (99% CI)** | **OR (99% CI)** | **OR (99% CI)** |
| **Total association** | 1.05 (1.03-1.07)* | 1.02 (1.01-1.03)* | 1.06 (1.03-1.09)* |
| **Direct association** | 1.04 (1.03-1.06)* | 1.01 (1.01-1.02)* | 1.05 (1.02-1.09)* |
| **Indirect association** | 1.01 (1.00-1.01)* | 1.00 (1.00-1.00)* | 1.01 (1.00-1.01)* |
|  |  |  |  |
|  | **Percentage** | **Percentage** | **Percentage** |
| **Mediating effects SEP and MetS remission** |  |  |  |
| Physical activity | -1.7 | -1.1 | -5.0 |
| Smoking | 3.7 | 2.8 | 4.6 |
| Alcohol intake | 5.6 | 9.1 | 8.1 |
| Diet quality | 5.3 | 4.3 | 6.3 |
| Health behaviors combined | 12.8 | 15.1 | 14.0 |
| OR: odds ratio; CI: confidence interval; SEP: socioeconomic position; MetS: metabolic syndrome; analyses controlled for years of education, household equivalized income, occupational prestige, age and sex at baseline, and time between baseline and second assessment; * p<0.01. | | | |
